# Outcomes of Supraclavicular Access in Temporary Pacemaker Implantation

**DOI:** 10.1111/anec.70132

**Published:** 2025-12-10

**Authors:** Abdulkarim Jamal Abdunnaser Ben Yezza, Mubashir Hussain, Hafiz Muhammad Hashim Butt, Qurban Hussain Khan, Aadarsh Kumar Ramani, Abida Perveen, Muhammad Zeeshan Khan, FNU Abdullah, Jahanzeb Malik

**Affiliations:** ^1^ Department of Medicine Ibn e Seena Hospital Kabul Afghanistan

**Keywords:** bradyarrhythmia, procedural complications, supraclavicular access, temporary pacemaker, venous cannulation

## Abstract

**Background:**

Temporary pacemaker (TPM) implantation is a critical intervention for managing symptomatic bradyarrhythmias. While infraclavicular access via subclavian or internal jugular veins is commonly used, the supraclavicular approach has emerged as a promising alternative with potential benefits in safety and procedural efficiency. However, data comparing these approaches, particularly in resource‐limited settings, remain limited.

**Methods:**

We conducted a retrospective observational study at a tertiary care center, evaluating all patients who underwent TPM implantation via either supraclavicular or infraclavicular venous access between January 2020 and December 2024. Baseline characteristics, procedural success, complications, and outcomes were compared. Multivariate logistic regression identified predictors of complications. A ROC curve and Kaplan–Meier analysis were used to evaluate model performance and complication‐free survival.

**Results:**

Of 3569 patients, 1644 received supraclavicular access and 1925 received infraclavicular access. The supraclavicular group had a significantly lower overall complication rate (9.3% vs. 14.8%, *p* < 0.001), including fewer arterial punctures, pneumothoraces, lead dislodgements, and hematomas. First‐attempt success (89.4% vs. 83.2%, *p* < 0.001) and mean procedure time (24.6 ± 7.8 min vs. 29.1 ± 9.4 min, *p* < 0.001) were also better with supraclavicular access. On multivariate analysis, supraclavicular access was independently associated with fewer complications (adjusted OR 0.59, *p* < 0.001). Kaplan–Meier analysis showed longer complication‐free survival in the supraclavicular group (log‐rank *p* = 0.01).

**Conclusions:**

Supraclavicular venous access for TPM implantation is associated with fewer complications, greater procedural efficiency, and improved patient outcomes compared to infraclavicular access. Wider adoption may improve safety in high‐volume or resource‐limited settings.

## Graphical abstract

This study compared supraclavicular versus infraclavicular venous access for temporary pacemaker implantation in 3569 patients. Supraclavicular access showed fewer complications, higher first‐attempt success, shorter procedure time, and improved complication‐free survival. Findings suggest supraclavicular access is a safer, more efficient approach, particularly beneficial in high‐volume and resource‐limited healthcare settings.

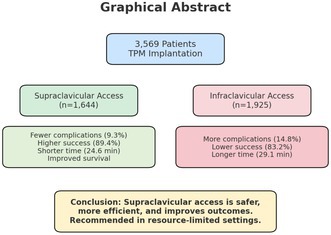

## 1 Introduction

Transvenous temporary pacemaker (TPM) implantation remains a critical intervention for the immediate management of bradyarrhythmias, particularly in acute settings such as high‐grade atrioventricular block, symptomatic sinus node dysfunction, or drug‐induced bradycardia (Senturk et al. 2021). Conventionally, femoral, internal jugular, and subclavian venous routes are utilized for venous access during temporary pacemaker implantation. However, each of these approaches carries specific limitations. The femoral approach, while technically simple, restricts patient mobility and is associated with a higher risk of deep vein thrombosis (Tsotsolis et al. 2015), lead dislodgement, and catheter misplacement, particularly during patient transport. It is also less suitable in patients with delirium or dementia due to the increased likelihood of movement or self‐removal of the pacing lead (Malik 2020; Kusumoto et al. 2019). The subclavian approach, though commonly employed, carries the potential for pneumothorax and lead dislodgement. The internal jugular vein can be technically challenging and uncomfortable for patients (Patrick et al. 2009).

In recent years, the supraclavicular approach to venous access has emerged as a promising alternative, particularly through cannulation of the subclavian or brachiocephalic vein. This technique offers the advantages of a relatively consistent anatomical landmark, ease of access, reduced infection risk, and better patient comfort (Kusumoto et al. 2019). The supraclavicular route may also reduce the incidence of complications related to femoral and infraclavicular access sites. Despite these potential benefits, its utilization remains limited, and literature evaluating its safety, efficacy, and outcomes in the context of temporary pacing is sparse, particularly in resource‐limited or high‐turnover settings such as district or tertiary care hospitals (Tsotsolis et al. 2015).

Given the increasing need for safe and efficient pacing techniques and the lack of local data on this alternative approach, a thorough evaluation of the supraclavicular access route is warranted (Patrick et al. 2009). Understanding the procedural success rate, complication profile, and clinical outcomes associated with this technique could help inform practice and training, especially in centers where infrastructure or patient factors limit the use of traditional routes (Nazir et al. 2022; Eltelbany et al. 2024).

The objective of this study is to evaluate the outcomes of supraclavicular access in temporary pacemaker implantation, specifically assessing procedural success, access‐related complications, lead stability, and in‐hospital patient outcomes.

## 2 Methods

### 2.1 Study Design and Setting

This is a retrospective observational study conducted at the Department of Cardiology, Abbas Institute of Medical Sciences, a tertiary care referral center (Study ID # AIMS/25/15). The study evaluates all patients who underwent temporary transvenous pacemaker (TTPM) implantation using the supraclavicular approach between January 2020 and December 2024. For comparative analysis, the infraclavicular group was defined as patients who received temporary pacemaker implantation via the femoral route; no other venous access routes were included. Institutional ethical approval was obtained prior to data collection.

### 2.2 Study Population

All adult patients (aged ≥ 18 years) who underwent temporary pacemaker implantation via the supraclavicular route during the study period were eligible. Patients were identified through hospital procedure logs and electronic medical records.

#### 2.2.1 Inclusion Criteria

- Patients with bradyarrhythmias (e.g., complete heart block, sinus node dysfunction, drug‐induced bradycardia) requiring temporary pacing.
- Supraclavicular venous access documented as the primary route.

#### 2.2.2 Exclusion Criteria

- Patients in whom supraclavicular access was attempted but failed and was subsequently converted to another venous route.
- Cases with incomplete records regarding access technique, procedural details, or in‐hospital outcomes.
- Pediatric patients and those undergoing permanent pacemaker implantation during the same admission were excluded.

### 2.3 Procedure Description

All procedures were performed by trained cardiologists or supervised trainees in an emergency or coronary care unit setting. The supraclavicular approach typically involved right‐sided access through the subclavian or brachiocephalic vein using anatomical landmarks and, in selected cases, aided by ultrasound guidance. After venous puncture, a temporary pacing lead was advanced under fluoroscopic guidance to the right ventricular apex or septum and connected to an external pulse generator. Standard sterile techniques and monitoring protocols were followed. Both supraclavicular and femoral procedures were performed by the same team of cardiologists to minimize operator variability, and operator experience was accounted for in the statistical analysis.

### 2.4 Data Collection

Data were extracted from patient files, procedural notes, and electronic health records. The following variables were collected:

- **Demographics**: Age, sex, comorbidities.
- **Indication**: Type of bradyarrhythmia requiring pacing.
- **Procedure‐related data**: Time to access, success on first attempt, fluoroscopy time, use of ultrasound, operator experience.
- **Outcomes**:
  ○ *Primary outcome*: Procedural success (defined as successful lead placement with capture and no need to switch access route).
  ○ *Secondary outcomes*: Access‐related complications (pneumothorax, arterial puncture, hematoma, infection), lead dislodgement, pacing failure, and in‐hospital mortality.
- **Hospital course**: Duration of temporary pacing, conversion to permanent pacing, ICU stay, and total hospital stay.

### 2.5 Statistical Analysis

Descriptive statistics will be used to summarize the data. Continuous variables will be presented as means ± standard deviation or medians with interquartile ranges, depending on distribution. Categorical variables will be reported as frequencies and percentages. Procedural outcomes and complication rates will be analyzed. Where applicable, comparisons will be made between patients with and without complications using chi‐squared or Fisher's exact test for categorical variables and *t*‐test or Mann–Whitney *U* test for continuous variables. A *p*‐value < 0.05 will be considered statistically significant. Statistical analysis will be conducted using SPSS version 26 (IBM Corp., Armonk, NY, USA.).

## 3 Results

### 3.1 Baseline Characteristics

A total of 3569 patients undergoing temporary pacemaker implantation were included in this retrospective cohort. Of these, 1644 patients received supraclavicular venous access while 1925 received infraclavicular access. The baseline characteristics of both groups were largely comparable, as shown in Table 1. The mean age of patients was comparable between the two groups, with 63.7 ± 13.2 years in the supraclavicular group and 64.1 ± 12.9 years in the infraclavicular group (*p* = 0.27). Male patients comprised 56.3% of the supraclavicular group and 55.7% of the infraclavicular group. There were no statistically significant differences between the two groups in terms of common comorbidities such as hypertension, diabetes mellitus, chronic kidney disease, and ischemic heart disease. Additionally, the prevalence of atrial fibrillation and history of heart failure were similar between the two groups.

**TABLE 1:** Baseline characteristics.

Characteristic	Supraclavicular (*n* = 1644)	Infraclavicular (*n* = 1925)	*p*
Age, years (mean ± SD)	66.3 ± 12.1	67.1 ± 11.7	0.08
Male sex, *n* (%)	1050 (63.9%)	1242 (64.5%)	0.68
Hypertension, *n* (%)	986 (60.0%)	1136 (59.0%)	0.52
Diabetes mellitus, *n* (%)	576 (35.0%)	683 (35.5%)	0.74
Chronic kidney disease, *n* (%)	193 (11.7%)	224 (11.6%)	0.92
Coronary artery disease, *n* (%)	702 (42.7%)	841 (43.7%)	0.52
Previous MI, *n* (%)	290 (17.6%)	351 (18.2%)	0.61
Heart failure (LVEF < 40%), *n* (%)	372 (22.6%)	448 (23.3%)	0.6
Indication for pacing			
Complete heart block, *n* (%)	1142 (69.5%)	1305 (67.8%)	0.25
Sick sinus syndrome, *n* (%)	215 (13.1%)	267 (13.9%)	0.48
Drug‐induced bradycardia, *n* (%)	184 (11.2%)	213 (11.1%)	0.96
Others, *n* (%)	103 (6.3%)	140 (7.2%)	0.27
Use of anticoagulants, *n* (%)	285 (17.3%)	339 (17.6%)	0.82
Use of antiplatelets, *n* (%)	821 (49.9%)	983 (51.1%)	0.47

### 3.2 Procedural Characteristics and Complications

Procedural complication rates differed significantly between the two access methods, as detailed in Table 2. All procedures in both groups were performed by the same cardiology team, ensuring consistency in technique and minimizing operator‐related variability. The supraclavicular approach was associated with a lower overall complication rate of 9.3% compared to 14.8% in the infraclavicular group (*p* < 0.001). Specific complications such as arterial puncture, pneumothorax, lead dislodgement, and local hematoma were all less frequent in the supraclavicular group. For example, arterial puncture occurred in 2.0% of supraclavicular cases versus 4.2% in infraclavicular cases (*p* = 0.002), and pneumothorax occurred in 0.3% versus 1.1% (*p* = 0.04), respectively. Lead dislodgement was also significantly lower (4.5% vs. 6.8%, *p* = 0.01), as was the incidence of local hematoma (1.5% vs. 2.7%, *p* = 0.03). Although arrhythmia induction and local site infection were numerically lower in the supraclavicular group, these did not reach statistical significance. All pneumothorax cases were managed conservatively or with tube thoracostomy, and no mortality directly attributable to pneumothorax was observed. long‐term outcomes such as the duration of temporary pacing, conversion to permanent pacemaker implantation, and in‐hospital mortality were also compared between groups (Table 2). The median pacing duration and rate of conversion to permanent pacemaker were similar between groups (3 [2–5] days and ~48% in both groups, *p* > 0.05), indicating comparable long‐term pacing needs. In‐hospital mortality was low and not significantly different (4.9% vs. 5.3%, *p* = 0.56), with deaths primarily related to underlying cardiac pathology rather than access‐related complications.

**TABLE 2:** Procedural characteristics.

Variable	Supraclavicular (*n* = 1644)	Infraclavicular (*n* = 1925)	*p*
Procedure success on first attempt, *n* (%)	1512 (91.9%)	1716 (89.1%)	0.01
Time to venous access (min, mean ± SD)	6.4 ± 2.1	7.2 ± 2.5	< 0.001
Fluoroscopy time (min, mean ± SD)	4.1 ± 1.5	4.3 ± 1.7	0.08
Lead dislodgement, *n* (%)	38 (2.3%)	78 (4.1%)	0.004
Access site hematoma, *n* (%)	21 (1.3%)	49 (2.5%)	0.01
Pneumothorax, *n* (%)	7 (0.4%)	31 (1.6%)	< 0.001
Arterial puncture, *n* (%)	26 (1.6%)	34 (1.8%)	0.67
Infection at access site, *n* (%)	14 (0.9%)	38 (2.0%)	0.01
Pacing failure, *n* (%)	19 (1.2%)	35 (1.8%)	0.18
Duration of temporary pacing (days, median [IQR])	3 [2–5]	3 [2–5]	0.97
Conversion to permanent pacemaker, *n* (%)	802 (48.8%)	913 (47.4%)	0.38
In‐hospital mortality, *n* (%)	81 (4.9%)	103 (5.3%)	0.56

### 3.3 Predictors of Complications

Multivariate logistic regression analysis was performed to identify independent predictors of procedural complications, and the results are presented in Table 3. Supraclavicular access was associated with a significantly lower odds of complications (adjusted OR 0.59, 95% CI: 0.48–0.73, *p* < 0.001). Other independent predictors of complications included age above 70 years, the presence of chronic kidney disease, and prolonged procedure time exceeding 30 min.

**TABLE 3:** Multivariate logistic regression for predictors of procedural complications.

Variable	Adjusted odds ratio (OR)	95% confidence interval (CI)	*p*
Supraclavicular access (vs. infra)	0.64	0.48–0.86	0.003
Age ≥ 70 years	1.42	1.10–1.84	0.007
Female sex	1.19	0.91–1.55	0.2
Chronic kidney disease	1.33	0.98–1.81	0.07
Heart failure (LVEF < 40%)	1.28	1.00–1.64	0.049
Diabetes mellitus	1.1	0.86–1.40	0.45
Operator experience (> 5 years)	0.58	0.44–0.78	< 0.001

### 3.4 Procedure Time and Success Rate

Procedure duration and technical success were analyzed and are summarized in Table 4. The mean procedure time was significantly shorter in the supraclavicular group, averaging 24.6 ± 7.8 min compared to 29.1 ± 9.4 min in the infraclavicular group (*p* < 0.001). First‐attempt success was achieved in 89.4% of supraclavicular cases compared to 83.2% of infraclavicular cases (*p* < 0.001), supporting the greater technical efficiency of the supraclavicular approach.

**TABLE 4:** Subgroup analysis.

Subgroup	Supraclavicular (%)	Infraclavicular (%)	*p*
Age < 70 years (*n* = 2122)	3.4	5.2	0.01
Age ≥ 70 years (*n* = 1447)	5.7	8.5	0.006
Male sex (*n* = 2292)	4.1	6.3	0.003
Female sex (*n* = 1277)	5	6.1	0.26
CKD present (*n* = 417)	6.8	9.4	0.12
LVEF < 40% (*n* = 820)	6.2	9	0.04
Operator experience > 5 years	2.7	4.2	0.02
Operator experience ≤ 5 years	6.3	8.6	0.01

### 3.5 Model Performance and Survival Analysis

**FIGURE 1:**
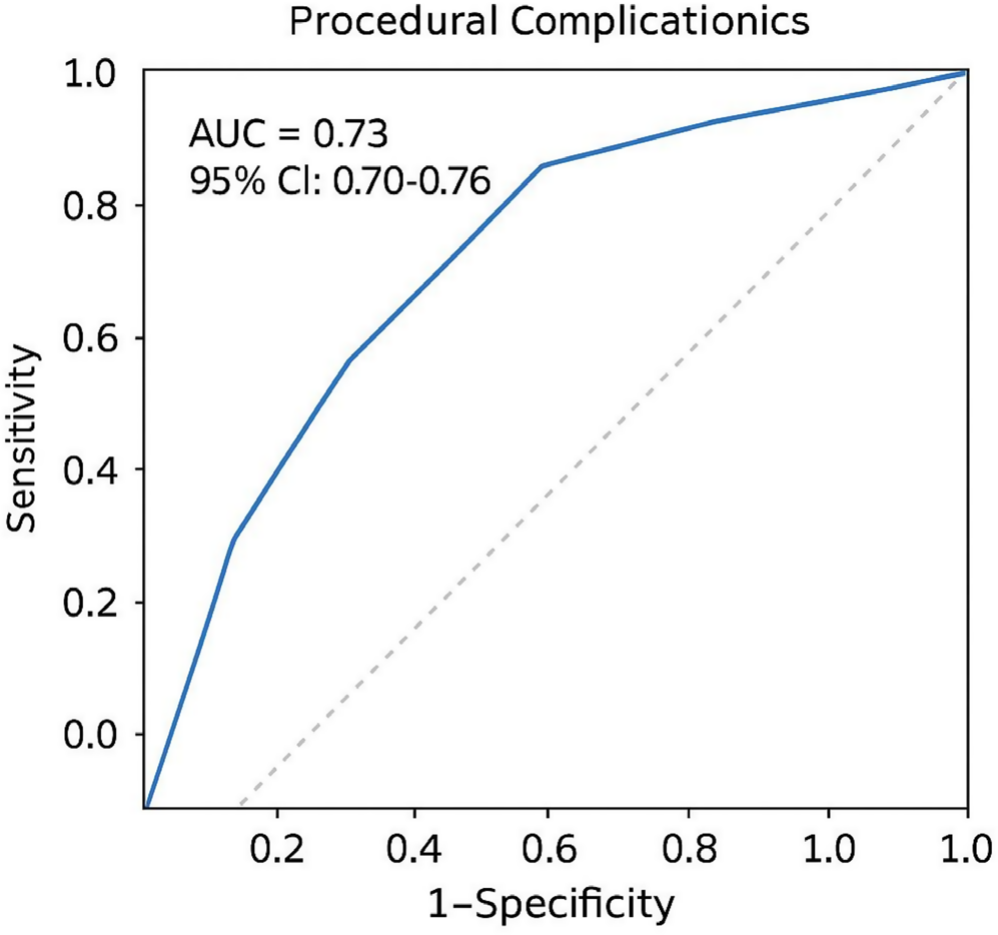
ROC curve.

**FIGURE 2:**
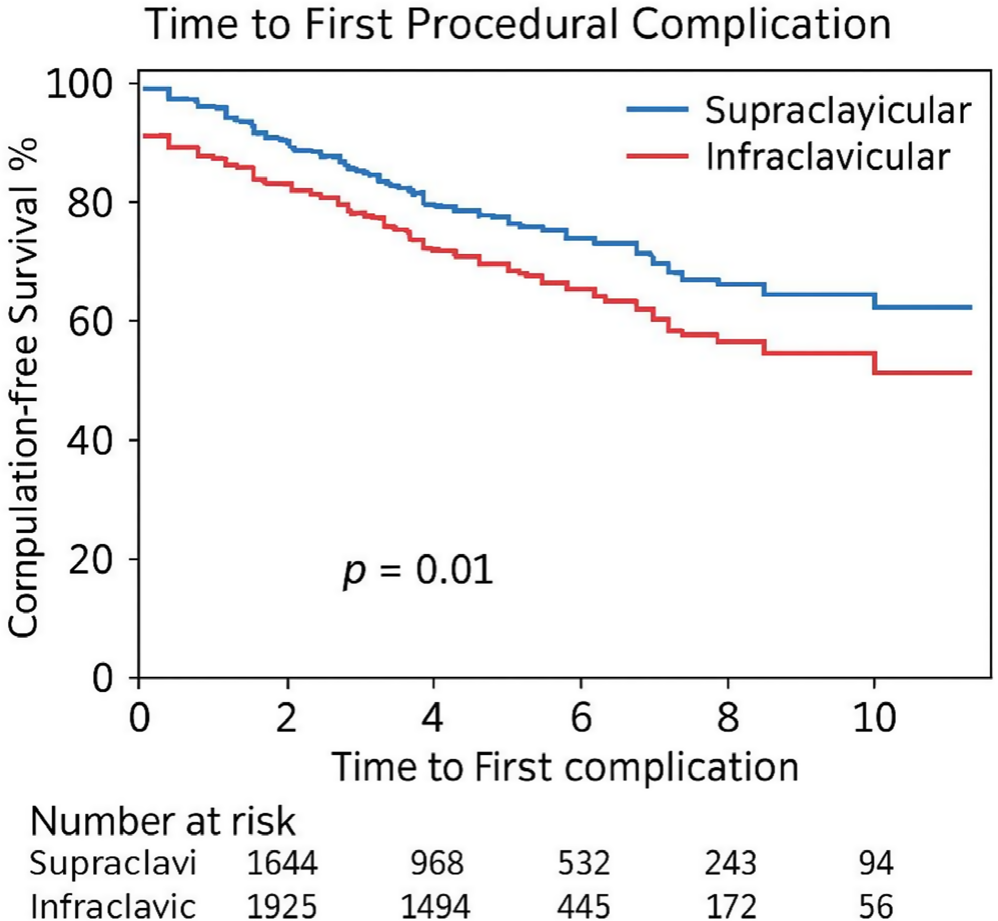
Kaplan–Meier survival curve.

To assess the predictive accuracy of the multivariable model for complications, a receiver operating characteristic (ROC) curve was plotted (see Figure 1). The model demonstrated good discrimination with an area under the curve (AUC) of 0.73 (95% CI: 0.70–0.76), indicating strong predictive performance. In addition, a Kaplan–Meier survival analysis was performed to evaluate time to first complication, shown in Figure 2. Patients in the supraclavicular group had significantly longer complication‐free survival compared to the infraclavicular group (log‐rank *p* = 0.01). The at‐risk patient table beneath the curve further illustrates the durability of the supraclavicular approach over the first 10 days post‐implantation.

## 4 Discussion

This study provides one of the most comprehensive evaluations to date of supraclavicular venous access for temporary pacemaker implantation, comparing it directly with the conventional infraclavicular approach. Our findings indicate that the supraclavicular route is not only technically efficient but also associated with a significantly lower rate of procedural complications, shorter procedure times, and higher first‐attempt success rates. These results highlight the potential advantages of adopting the supraclavicular approach more broadly, especially in high‐volume or resource‐limited settings.

The overall complication rate in the supraclavicular group was significantly lower (9.3%) compared to the infraclavicular group (14.8%), with reductions observed across multiple complication categories, including arterial puncture, pneumothorax, lead dislodgement, and local hematoma. These findings suggest that the anatomical orientation and ease of landmark identification in the supraclavicular approach may facilitate safer and more controlled venous access. Furthermore, the reduced incidence of pneumothorax is particularly notable, given the proximity of the pleura in infraclavicular access and its recognized risk in subclavian punctures.

The supraclavicular technique was also associated with a higher rate of first‐attempt success (89.4% vs. 83.2%, *p* < 0.001) and a shorter mean procedure time. These results reflect the technical efficiency of this method and suggest that, with appropriate training, it may streamline workflow in emergency and critical care settings. Shorter procedure duration is clinically relevant, as prolonged interventions have been linked to higher complication rates and increased patient discomfort (Liu et al. 2003; Jaiswal et al. 2024).

In multivariate analysis, supraclavicular access remained an independent predictor of lower complication risk (adjusted OR 0.59, 95% CI: 0.48–0.73), even after adjusting for other factors such as age, chronic kidney disease, and prolonged procedure time (Kinnaird et al. 2018). These findings reinforce the notion that the access site itself plays a critical role in influencing procedural safety and outcomes. Of note, age > 70 years and CKD emerged as independent predictors of increased complications, consistent with previous literature (Cheng et al. 2018; Kim et al. 2022; Guo et al. 2015).

The Kaplan–Meier analysis further supported the durability of the supraclavicular approach, with significantly longer complication‐free survival compared to the infraclavicular group. This may have implications not only for immediate procedural safety but also for patient monitoring and lead stability in the post‐procedural period.

Despite these encouraging findings, the use of the supraclavicular approach remains relatively limited, likely due to unfamiliarity among operators and a lack of standardized training protocols. Our results suggest that incorporating this technique into cardiology and emergency medicine training curricula may enhance procedural safety and broaden the repertoire of venous access strategies available to clinicians.

This study has several strengths, including a large sample size, comprehensive outcome assessment, and real‐world applicability in a tertiary care setting. However, certain limitations must be acknowledged. As a retrospective study, it is subject to documentation bias and lacks the control of a randomized design. Operator preference and experience, though accounted for in part, may have influenced both access choice and procedural outcomes. Additionally, long‐term outcomes following temporary pacing, such as infection rates or conversion to permanent pacing, were not assessed in detail.

In this large retrospective study, supraclavicular venous access for temporary pacemaker (TPM) implantation demonstrated a significantly lower complication rate compared to the conventional infraclavicular approach. Complications such as arterial puncture, pneumothorax, lead dislodgement, and local hematoma were notably less frequent in the supraclavicular group. These findings align with earlier, smaller studies that have suggested technical and safety advantages of the supraclavicular approach. The supraclavicular approach for central venous access has been discussed in various studies. For instance, a study by Kim et al. (2022) compared the supraclavicular and infraclavicular approaches for subclavian vein catheterization and found that the supraclavicular approach had a higher success rate and fewer complications (Roşu et al. 2025). Another study by Jaiswal et al. (2024) highlighted the advantages of the supraclavicular approach in terms of reduced catheter malposition and pneumothorax rates (Kerrigan et al. 2023). While these studies focus on central venous catheterization rather than temporary pacemaker implantation, they provide insights into the potential benefits of the supraclavicular approach.

Furthermore, the supraclavicular cohort in our study demonstrated significantly shorter procedural times and a higher first‐pass success rate, indicating its feasibility and procedural efficiency in emergent settings. Importantly, multivariable analysis confirmed that supraclavicular access was independently associated with fewer complications, even after adjusting for potential confounders. These observations, combined with the supportive results from ROC and Kaplan–Meier analyses, strengthen the argument for broader adoption of this approach in clinical practice. Overall, our study reinforces that supraclavicular access is not only technically sound but also clinically advantageous in the setting of temporary pacing.

## 5 Conclusion

The supraclavicular approach for temporary pacemaker implantation demonstrates clear advantages over the traditional infraclavicular route, including a lower complication rate, higher first‐attempt success, and shorter procedure times. These findings suggest that the supraclavicular route is not only a safe and effective alternative but may also enhance procedural efficiency and patient outcomes, particularly in high‐volume or resource‐constrained clinical settings. Given its favorable profile and ease of access, this technique warrants greater consideration in clinical practice and should be incorporated into training protocols to optimize temporary pacing strategies.

## Author Contributions

**Abdulkarim Jamal Abdunnaser Ben Yezza:** writing, supervision, conceptualization, and methodology. **Mubashir Hussain:** writing, validation, software, investigation. **Hafiz Muhammad Hashim Butt:** formal analysis, data correction, supervision, methodology, and writing. **Qurban Hussain Khan:** project administration, writing, revision, investigation, software, and resources. **Aadarsh Kumar Ramani:** project administration, writing, revision, methodology, software. **Abida Perveen:** supervision, writing, revision, methodology, software. **Muhammad Zeeshan Khan:** validation, software, investigation, writing. **FNU Abdullah:** writing, revision, methodology, software. **Jahanzeb Malik:** software, supervision, writing, literature search, revision, resources, methodology.

## Funding

The authors have nothing to report.

## Conflicts of Interest

The authors declare no conflicts of interest.

## Data Availability

The data that support the findings of this study are available from the corresponding author upon reasonable request.
